# Non-Targeted and Targeted Analysis of Organic Micropollutants in Agricultural Soils Across China: Occurrence and Risk Evaluation

**DOI:** 10.3390/toxics14010025

**Published:** 2025-12-25

**Authors:** Caifei Xu, Yang Qiu, Weisong Chen, Nan Liu, Xingjian Yang

**Affiliations:** 1 Key Laboratory of Arable Land Conservation (South China), Ministry of Agriculture, College of Natural Resources and Environment, South China Agricultural University, Guangzhou 510642, China; 2 Guangdong Province Key Laboratory for Agricultural Resources Utilization, College of Natural Resources and Environment, South China Agricultural University, Guangzhou 510642, China; 3 Guangdong Research Center for Agricultural Soil Pollution Prevention and Control Engineering Technology, College of Natural Resources and Environment, South China Agricultural University, Guangzhou 510642, China; 4 Joint Institute for Environment & Education, College of Natural Resources and Environment, South China Agricultural University, Guangzhou 510642, China

**Keywords:** occurrence, spatial distribution, cultivation modes, crop types, ecological risks

## Abstract

Organic micropollutants in agricultural soils pose significant ecological and health risks. This study conducted the first large-scale, integrated non-targeted screening and targeted analysis across China’s major food-producing regions. Using high-resolution mass spectrometry, 498 micropollutants were identified, including pesticides, industrial chemicals, pharmaceuticals, personal care products, food additives, natural products, and emerging contaminants. Spatial analysis revealed strong correlations in pesticide detections between Henan and Hebei, as well as between Hebei and Shandong, indicating pronounced regional similarities in pesticide occurrence patterns. Concentrations of 50 quantified micropollutants showed clear spatial variability, which was associated with precipitation, water use, and agricultural output, reflecting climate–agriculture–socioeconomic synergies. Greenhouse soils accumulated higher micropollutant levels than open fields, driven by intensive agrochemical inputs, plastic-film confinement, and reduced phototransformation. Co-occurrence patterns indicated similar pathways for personal care products, industrial chemicals, and pesticides, whereas natural products and pharmaceuticals showed lower levels of co-occurrence due to crop-specific exudates, fertilization, and rainfall-driven leaching. Among cropping systems, orchard soils had the highest micropollutant accumulation, followed by paddy and vegetable soils, consistent with frequent pesticide use and minimal tillage. Risk quotients indicated moderate-to-high ecological risks at over half of the sites. These results reveal complex soil pollution patterns and highlight the need for dynamic inventories and spatially differentiated, crop- and system-specific mitigation strategies.

## 1. Introduction

With the continued growth of the global population, pressure to increase food production remains intense [1]. In response, greenhouse agriculture has expanded rapidly as an intensive cultivation strategy, particularly in areas where traditional open-field farming is limited [2]. However, both conventional and greenhouse farming systems have imposed significant environmental stresses, contributing to widespread soil degradation, which now affects approximately 25% of the global soils [3]. China exemplifies these challenges, exhibiting distinct regional contamination patterns driven by varied land-use, climatic conditions, and agricultural and industrial activities. Reported pollutants range from polycyclic aromatic hydrocarbons in Northeast China to organochlorine pesticides and phthalate esters in northern and central regions, as well as polychlorinated biphenyls in eastern and southern provinces [4]. These issues underscore the urgent need to better understand the mechanisms of soil pollutant accumulation and behavior across different agricultural systems, in support of sustainable land management and food security.

Organic micropollutants pose escalating threats to the environment under accelerating urbanization and industrialization pressures. The pollutant spectrum spans diverse anthropogenic chemicals, such as pesticides (e.g., fungicides and herbicides), industrial chemicals (e.g., flame retardants and dyes), microplastics, pharmaceuticals, personal care products, and food additives [5]. Soil functions as a critical environmental sink for organic micropollutants, with contamination originating from agrochemical application [6], atmospheric deposition [7], manure fertilization [8], and reclaimed water irrigation [9]. The prolonged accumulation of these substances in soil systems generates persistent, multi-dimensional contamination networks that gradually destabilize ecosystem stability. They perturb critical biotic interactions by altering microbial community composition and functional diversity [10], triggering trophic cascades that compromise organismal survival, reproductive fitness, and ultimately human health through food-chain biomagnification [11].

While extensive studies have characterized the occurrence and spatiotemporal dynamics of specific micropollutant classes in environmental systems [12], conventional targeted approaches remain constrained by predefined compound libraries. In contrast, non-targeted screening represents a paradigm shift, employing high-resolution mass spectrometry (HRMS)-based workflows to simultaneously detect known, unrecognized, and emerging micropollutants. This technique has demonstrated broad applicability across diverse environmental matrices, including water [13], soils [14], atmospheric dust [15], and sediments [16]. Nevertheless, non-targeted screening in large-scale soil studies, particularly in China’s major food-producing regions, remains limited. Profound heterogeneity in climate, agronomic practices, and soil properties across these regions may drive significant divergence in micropollutant profiles that require further characterization [17]. Furthermore, China’s rapid greenhouse agriculture expansion can introduce distinct contamination dynamics compared to open-field systems. Controlled-environment cultivation modifies key micropollutant drivers through changed agrochemical application patterns [18], altered hydrological dynamics [2], and altered atmospheric deposition fluxes under plastic-film enclosures [19]. These operational differences likely generate unique micropollutant fingerprints in greenhouse soils, necessitating systematic non-targeted screening to inform precision soil management strategies.

Despite advances in targeted and non-targeted analyses, large-scale soil studies in China’s major food-producing regions remain limited, particularly in comparing greenhouse and open-field cultivation systems. To address these knowledge gaps, this study aimed to (i) systematically characterize the occurrence and spatial distribution of organic micropollutants across eight key agricultural regions in China, (ii) quantify 50 representative substances, including pesticides, pharmaceuticals, and industrial chemicals, and (iii) evaluate their ecological risks in relation to cultivation systems and crop types. By integrating non-targeted screening with targeted quantification, this research provides a comprehensive assessment of soil micropollutant profiles and their potential environmental impacts, offering novel insights for precision soil management and sustainable agriculture strategies.

## 2. Materials and Methods

### 2.1. Chemicals

Analytical standards used in this study (purity: 95.7–99.9%) were purchased from Alta Scientific Co. (Tianjin, China). Formic acid (HPLC grade) and ammonium acetate (analytical grade) were obtained from DiKMA Technologies Inc. (Foothill Ranch, CA, USA) and Kermel Chemical Reagent Co. (Tianjin, China), respectively. Methanol and water (HPLC and LC-MS grade) were purchased from Fisher Scientific (Pittsburgh, PA, USA).

### 2.2. Sample Collection and Pretreatment

In this study, 73 surface soil samples (0–30 cm depth) were collected in 2019 from eight major food-producing regions across China: Heilongjiang (*n* = 8), Jilin (*n* = 6), Liaoning (*n* = 6), Hebei (*n* = 6), Henan (*n* = 9), Shandong (*n* = 12), Guangdong (*n* = 18), and Tianjin (*n* = 8). This sample size, while focused for a national-scale study, was strategically determined based on a pre-existing national sampling framework originally designed for steroid hormone analysis, ensuring coverage of critical variations in cropping systems, climatic conditions, and land-use types [20]. All Guangdong samples originated from open-field systems (vegetable, rice, and orchard land uses) as ~87% of China’s greenhouses are concentrated in the major agricultural regions of northwest China, the Huang-Huai-Hai, Bohai Rim, and Yangtse River Delta [21]. The remaining 55 samples comprised 28 greenhouse soils and 27 open-field soils from other regions, representing diverse cropping systems such as vegetables, maize, pulses, and wheat. Sampling locations are documented in Table S1. The final sample number was considered appropriate for the current non-targeted screening, which required extensive sample preparation, high-resolution instrumental analysis, and substantial data processing. Accordingly, the sampling strategy focused on capturing pollutant diversity across representative agricultural settings rather than achieving high-density geographic coverage.

At each sampling site, five subsamples were collected and homogenized to create composite samples, wrapped in aluminum foil, and stored in polythene bags. Samples were transported to the laboratory under dark conditions, freeze-dried (−40 °C), sieved (2 mm), and stored at −20 °C in a light-protected refrigerator until analysis. All samples were analyzed within three months after collection to minimize potential changes in chemical composition. Soil aliquots (2.00 g) were weighed into glass centrifuge tubes and spiked with eight isotopically labeled internal standards (ISTD, atrazine-D_5_, carbendazim-D_3_, hexazinone-D_6_, metalaxyl-D_6_, thiamethoxam-D_3_, progesterone-D_9_, carbamazepine-D_10_, and 5-methylbenzotrazole-D_6_). Extraction followed an optimized protocol from our prior work [20]. The detailed extraction process is outlined in Text S1, and the final extracts were reconstituted for instrumental analysis.

### 2.3. Instrumental Analysis

HRMS analysis was performed using an Agilent 1290 Infinity II high-performance liquid chromatograph (HPLC) coupled to an Agilent 6545B quadrupole time-of-flight mass spectrometer (QTOF-MS). Chromatographic separation was achieved on a ZORBAX Eclipse Plus C18 analytical column (2.1 × 100 mm, 1.8 μm) equipped with an Eclipse Plus C18 guard column (2.1 × 5 mm, 1.8 μm). Column temperature was maintained at 45 °C, with a 1 μL injection volume. The mobile phase, elution gradient, and data acquisition procedure followed established protocols [22], with detailed information shown in Text S2. Data acquisition included electrospray ionization positive (ESI+) and negative modes (ESI-). Targeted quantification of 50 micropollutants was conducted on an Agilent 1290 Infinity II HPLC system interfaced with an Agilent 6495C triple quadrupole mass spectrometer (TQ-MS). A ZORBAX Eclipse Plus C18 analytical column (2.1 × 50 mm, 1.8 μm) was employed under identical flow rates and gradient conditions as the HPLC-QTOF-MS method to ensure analytical consistency (Text S2).

### 2.4. Quality Assurance and Quality Control

For the HPLC-QTOF-MS analysis, mass accuracy was calibrated by intermittently injecting a leucine enkephalin calibration solution at 15 s intervals during each sample measurement. An ISTD control solution (nafcillin-D_5_, coumaphos-D_10_, amoxicillin-D_4_, cephalexin-D_5_, and ceftiofur-D_3_) and a control group (methanol blank and ISTD blank) were analyzed after every 15 samples to monitor retention time (RT) stability. RT (<0.1 min) and mass (5 ppm) deviations of the ISTDs were within acceptable limits throughout the analyses. Quantitative analysis of 50 micropollutants employed an internal standard method, which demonstrated excellent linearity (R^2^ > 0.99). To verify the efficiency of the sample pretreatment procedure, recovery tests were performed by spiking 10 μL of a 1 ppm mixed standard solution into 2 g of quartz sand as an inert surrogate matrix, corresponding to a fortification level of 5 ng/g. The recoveries of pesticides, pharmaceuticals, and industrial chemicals were in the ranges of 70.0–138%, 87.0–104%, and 90.0–108%, respectively. Method reproducibility was estimated by performing triplicate quantitative analyses on soil samples from each experimental batch, yielding relative standard deviations of 3–17% for all target analytes. The method detection limits (MDLs) and quantification limits (MQLs) of the micropollutants were estimated based on the lowest standard concentrations spiked into the SPE extracts with signal-to-noise (S/N) ratios of 3 and 10, respectively. The MDLs and MQLs for the 50 micropollutants in the soils ranged from 0.001 to 0.077 ng/g and from 0.003 to 0.257 ng/g, respectively. Detailed information on the recoveries, MDLs, and MQLs of the target micropollutants is shown in Table S2.

### 2.5. Data Prioritization and Micropollutant Identification

The raw format data were converted to mzML format using Msconvert 3.0 and subsequently processed using the PyHRMS program for feature extraction, filtering, and alignment [23]. Features with mass intensities greater than 500 and peak areas at least five times greater than those found in blank groups were retained for further analysis. The retained features were then matched by three major databases: (i) an in-house database containing basic information (e.g., *m*/*z* values, MS^2^ fragments, and RTs) for over 2000 micropollutants; (ii) Massbank of North America, which holds over 145,000 LC-MS spectra (https://massbank.us/downloads, accessed on 27 June 2025); and (iii) NORMAN Suspect List Exchange (http://www.norman-network.com/nds/SLE, accessed on 2 July 2025). The identification of compounds was conducted according to the confidence hierarchy established by Schymanski [24]. Level 1 micropollutants were confirmed through strict validation, requiring chromatographic consistency (RT deviation < 0.1 min), precise mass spectral matching (MS^1^ error < 10 ppm) against authentic standards, and the identification of at least one diagnostic MS^2^ fragment. Level 2 micropollutants were identified based on spectral library matching, requiring a MS^1^ (MS^1^ error < 10 ppm) and a minimum of two concordant MS^2^ fragments in the absence of authentic standards. Micropollutants matching only by RT and MS^1^, but failing to yield MS^2^ data, were assigned to level 3.

All subsequent statistical analyses and data visualization were performed using Origin 2022 (OriginLab), Python 3.11.3, and ArcGIS 10.5.

### 2.6. Ecological Risk Assessment

Due to the lack of standardized soil ecotoxicity benchmarks and consistent assessment methodologies for pharmaceuticals and industrial chemicals, ecological risk assessments were restricted to pesticides. This focus was further justified as pesticide input constituted the predominant source of micropollutants in intensively managed agricultural soils, ensuring methodological comparability and relevance to the studied systems. The ecological risks were evaluated using the risk quotient (*RQ*) approach [25] based on the following equation:

(1) $${\mathrm{RQ}}_{i}\;=\;{\mathrm{MEC}}_{\mathrm{soil}}/{\mathrm{PNEC}}_{\mathrm{mss}}$$

Here, *MEC*_soil_ denotes the measured concentration (mg/kg) of micropollutant i in the soils. *PNEC*_mss_ represents the predicted-no-effect concentration of a micropollutant derived from toxicity data for the most sensitive organisms. *PNEC*_mss_ values were calculated using either no-observed-effect concentrations (*NOEC*) or median lethal concentrations (*LC*_50_) divided by assessment factors (*AF*), as detailed in Table S3. Following established methodologies [25], acute evaluations for micropollutants employed the lowest *LC_50_* divided by an *AF* of 1000, whereas chronic evaluations used *NOEC* values divided by an *AF* of 10. Chronic toxicity data were prioritized where available. Toxicological datasets were sourced from three authoritative databases: (i) the pesticide properties database (PPDB, https://sitem.herts.ac.uk/aeru/ppdb, accessed on 5 July 2025); (ii) the European Food Safety Authority (EFSA, https://www.efsa.europa.eu, accessed on 7 July 2025); and (iii) the U.S. Environmental Protection Agency ECOTOX knowledgebase. The calculated *RQ*s for micropollutants were classified into four categories: negligible risk (*RQ* < 0.01), low risk (0.01 ≤ *RQ* < 0.1), medium risk (0.1 ≤ *RQ* < 1), and high risk (*RQ* ≥ 1) [26].

The cumulative ecological risks were evaluated based on the following equation [27]:

(2) $$\sum {\mathrm{RQ}}_{\mathrm{site}}=\;\sum_{i\;=\;1}^{n}{\mathrm{RQ}}_{i}$$

Σ*RQ*_site_ represents the cumulative risk for a sampling site, calculated as the sum of individual *RQ*_i_ for all detected pesticides at that location. Cumulative risks were classified into four categories: negligible risk (Σ*RQ*_site_ *<* 0.01), low risk (0.01 ≤ Σ*RQ*_site_ < 0.1), moderate risk (0.1 ≤ Σ*RQ*_site_ < 1), and high risk (Σ*RQ*_site_ ≥ 1) [27].

To quantify the relative contribution of individual micropollutants to cumulative mixture risks, the following equation was applied:

(3) $$\mathrm{Contribution}\;=\;{\mathrm{RQ}}_{i}/\sum {\mathrm{RQ}}_{\mathrm{site}}\;\times \;100$$

## 3. Results

### 3.1. Comprehensive Identification of Organic Micropollutants in the Soils

**Figure 1:**
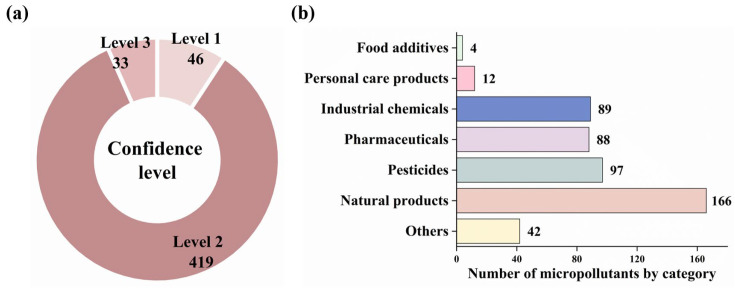
Micropollutants were detected in the soils (*n* = 498) from major food-producing regions in China. (**a**) Confidence levels of identified micropollutants: level 1, level 2, and level 3. (**b**) Categories of micropollutants with corresponding numbers. The “Others” denotes micropollutants without clear identity, functions, or applications.

In this study, non-targeted screening detected 7678 features under ESI+ mode and 6245 features under ESI- mode across the soil samples. Through comprehensive database matching, 498 micropollutants were identified and classified into three confidence levels (Figure 1a): level 1 (*n* = 46), level 2 (*n* = 419), and level 3 (*n* = 33). The mass spectra of some representative substances are shown in Figure S1. These compounds were then categorized to elucidate their relationship with anthropogenic activities. Natural products constituted the dominant category (*n* = 166), followed by pesticides (*n* = 97), industrial chemicals (*n* = 89), and pharmaceuticals (*n* = 88), with personal care products (*n* = 12) and food additives (*n* = 4) constituting minor components (Figure 1b). Comprehensive compound data are documented in Table S4. Based on the availability of laboratory reference standards, quantitative analysis was performed on 50 selected micropollutants, including 45 pesticides, 3 pharmaceuticals, and 2 industrial chemicals. Quantified concentrations spanned ND–10.2 ng/g for industrial chemicals, ND–45.1 ng/g for pharmaceuticals, and ND–2786 ng/g for pesticides (Table S5).

#### 3.1.1. Pesticides

The 97 identified pesticides comprised 20 herbicides, 35 fungicides, 36 insecticides, and 6 pesticide metabolites (Figure S2). The two most frequently detected pesticides were identified as 2,7,8,9-tricyclazole (detection frequency, DF = 69.7%) and chlorantraniliprole (DF = 60.3%). 2,7,8,9-tricyclazole, a systemic fungicide, exhibits persistent efficacy against rice blast disease; however, its environmental persistence raises ecological concerns. One study indicated that this compound can induce hepatocyte apoptosis in zebrafish and disrupt glucolipid metabolism and energy homeostasis, potentially impairing liver development and function in vertebrates [28]. Chlorantraniliprole, an o-aminobenzamide insecticide targeting lepidopteran pests, has been shown to impair the growth and metamorphosis of silkworm larvae even at sublethal concentrations [29]. Of the 45 pesticides quantified, 24 were fungicides, 13 were insecticides, and 8 were herbicides. Among these, fenuron exhibited the highest mean concentration across all 73 soil samples (range: ND–1029 ng/g; mean: 107 ng/g). Following its extensive agricultural application, fenuron can enter the food chain via crop absorption. Direct exposure can cause eye, skin, and respiratory tract irritation, while ingestion of high doses may induce gastrointestinal symptoms or even methaemoglobinaemia [30]. One study [31] reported that 57% of fenuron applied to high-calcium soils leached through soil columns, highlighting significant transport potential.

Notably, several pesticides explicitly banned in China were detected, such as furadan, triazophos, fipronil, and chlorpyrifos, along with prohibited pesticide metabolites, such as methomyl sulphoxide, fipronil sulfide, fipronil desulfurised sulphone, and fipronil sulphone. Chlorpyrifos (an organophosphate insecticide), detected only in two Guangdong orchard soils (53.6–2786 ng/g), exceeded the maximum level of 5.58 ng/g documented in the paddy soils from the middle reaches of the Yangtze River [32]. This compound disrupts blood glucose regulation, promotes obesity development, and stimulates cell division in human breast cancer cells [33]. Classified as a highly hazardous pesticide by the United States Environmental Protection Agency (U.S. EPA), chlorpyrifos nevertheless continued to be illegally applied in many countries such as India and China [34]. Global usage exceeded 200,000 metric tons in 2015 alone, with projected increases despite known risks [35].

Additionally, our analysis identified five pesticides banned in the European Union, including clothianidin, propiconazole, (Z)-dimethylmorpholine, S-metolachlor, and isocarbazone. Clothianidin is a typical neonicotinoid insecticide that acts as a potent neurotoxin, targeting insect acetylcholine receptors to cause rapid paralysis and death. However, its residues in aquatic environments may harm non-target organisms like aquatic arthropods [36]. S-metolachlor, a selective chloroacetamide herbicide widely used on major crops, persists in soil, damaging soil ecosystems. Its uptake by crops poses health risks, including endocrine disruption and potential carcinogenicity [37]. Notably, our analytical workflows detected certain pesticides rarely documented in scientific literature, such as pyroquilon, ethirimol, and coumaphos oxon.

To provide context for the pesticide levels observed in this study, we compared our results with representative global studies on pesticides in agricultural soils (Table S6). The cumulative pesticide concentrations observed here (ND–2786 ng/g) were comparable to the ranges reported for European agricultural soils (ND–2870 ng/g) [38], while being higher than the ranges reported for arable soils in the Czech Republic (2–269 ng/g) [39] and peach orchard soils in China (1.05–327 ng/g) [17]. These differences may partly reflect the intensive pesticide application practices in the study region, as well as methodological differences among studies, particularly regarding the number and types of pesticides included in quantitative analysis.

#### 3.1.2. Pharmaceuticals

A total of 88 pharmaceuticals were identified in the soils, which mainly included 13 antibiotics, 8 cardiovascular drugs, 6 hormones, 6 psychotropic drugs, 6 anticancer agents, 6 analgesic drugs, 5 anticoagulants, 2 respiratory drugs, and 1 antidiabetic drug (Figure S2). Butyrolactone I (DF = 76.8%) and Deprenyl (DF = 34.2%) were the two most frequently detected pharmaceuticals. Butyrolactone I, a metabolite of *Aspergillus africanus* variant (IFO 8355), exhibits significant antiproliferative activity against colon, pancreatic, lung, and prostate cancer cells [40]. Deprenyl, a selective monoamine oxidase-B inhibitor used in Parkinson’s therapy, has recently demonstrated efficacy in alleviating depressive symptoms in major depressive disorder [41]. Among the quantified pharmaceuticals, nicotine was detected at relatively high concentrations (ND–45.1 ng/g). As a pharmacologically active alkaloid originally derived from *Nicotiana* species, its presence in environmental matrices is primarily anthropogenic, resulting from the manufacture, use, and disposal of tobacco products. This study also uncovered some previously underreported compounds, such as 1-(2-chlorophenyl) piperazine, 5-carboline, dosulepin (three psychotropic drugs), thielavin B, 8-hydroxyquinoline, and albocycline (three antibiotics and antifungals).

#### 3.1.3. Industrial Chemicals

A total of 89 industrial chemicals were identified in the soils, including 47 chemical intermediates, 12 dyes, 12 plasticizers/flame retardants, 11 surfactants, and 7 paints (Figure S2). Among these, 1,3-diphenylguanidine—a common vulcanization accelerator used in tire and rubber manufacturing [42]—was detected with a particularly high DF value (94.5%). Its prevalence was likely due to the widespread dispersion of tire wear particles and road dust via atmospheric deposition, surface runoff, and irrigation with contaminated water [43]. In addition, its environmental persistence and strong affinity for soil particles can promote accumulation in soil matrices. In contrast, the two quantified industrial chemicals, triisobutyl phosphate and octhilinone, were found at low concentrations (<10 ng/g). Notably, eight substances were classified as Group 2B carcinogens by the International Agency for Research on Cancer (IARC): dibutyl phthalate, 4-nitro-N-phenylaniline, 3,4,5-trichlorophenol, tris(2-chloroisopropyl) phosphate, nitrosomethylaniline, 4-aminoazobenzene, diethanolamine, and 4-nitrosodiphenylamine. Of particular concern was aniline (DF = 45.2%), an IARC Group 1 carcinogen extensively utilized in dyestuffs, rubber, and plastic manufacturing. This highly toxic pollutant induces early adverse effects upon entering living organisms, including the induction of methemoglobin formation, damage to red blood cells, and even spleen injury, congestion, and lipoatrophy [44]. In regulatory response, the Chinese standard (GB8978-1996) [45] enforced a strict maximum permissible concentration of 1.0 mg/L for aniline in wastewater discharges [46].

This study further identified six endocrine-disrupting industrial chemicals with environmental persistence and reproductive toxicity implications. These comprised three phthalates (dibutyl phthalate, diethyl phthalate, and benzyl butyl phthalate), one organic acid ester (tris(2-chloroisopropyl) phosphate), diethanolamine, and 4-nonylphenoxy-acetic acid, with DFs spanning 1.4% to 41.1%. Our investigation also revealed rarely documented industrial chemicals, including plasticizers such as triisobutyl phosphate and 1,3-diphenylguanidine, dyes like 2,6-di-tert-butyl-4-nitrophenol, and the paint compound 4-hydroxy-3-(3-methyl-2-butenyl)acetophenone. These findings significantly expanded the documented diversity of industrial pollutants in agricultural soils.

#### 3.1.4. Personal Care Products and Food Additives

Non-targeted analysis identified 12 personal care products across the seven regions studied (all except Tianjin City, Figure 1b). These compounds, which included 8 emulsifiers, 3 antimicrobials, and 1 detergent, exhibited generally low DFs across the soil samples. Triclocarban (DF = 8.2%), a widely used antimicrobial in soaps, hand sanitizers, and body washes, raises concerns due to developmental toxicity, antibiotic resistance promotion, and endocrine disruption [47], promoting its ban in consumer hand soaps by the U.S. Food and Drug Administration (FDA). Notably, the detergent sodium myristyl sulfate (DF = 20.6%) and the emulsifier lauramidopropylbetaine (DF = 8.2%) were detected in the soils; these compounds have rarely been reported in the literature. This study also detected four food additive analogs, including denatonium benzoate, a potent bitter flavoring agent found at 37% of the sampling sites.

#### 3.1.5. Natural Products

Natural products constituted the most abundant category of identified micropollutants (*n* = 166, Figure 1b), deriving from diverse sources such as plant and microbial metabolites and organic fertilizer degradation products. The detection of emodin (DF = 2.7%), a naturally occurring anthraquinone compound at site JL-5 and HLJ-8, is of concern given its demonstrated ecotoxicity exhibiting a median effective concentration (EC_50_) of 130 μg/L for *Daphnia similis* and an LC_50_ of 25 μg/L for zebrafish embryos [48]. Of particular regulatory significance was the identification of zearalenone in Shandong Province, an estrogenic mycotoxin produced by *Fusarium oxysporum* that can infect cereal grains, particularly wheat and maize [48,49]. This compound disrupts endocrine function through competitive binding to estrogen receptors, affecting endogenous estrogen levels, and causing reproductive disorders [50]. Chinese national standard (GB 2761-2017) [51] mandated a maximum limit of 60 µg/kg for zearalenone in cereals, while the European Food Safety Authority (EFSA) has established a tolerable daily intake (TDI) of 0.25 µg/kg body weight [52].

### 3.2. Spatial Distribution of Micropollutants Across Different Regions

#### 3.2.1. Spatial Distribution of Characteristic Features

Spatial analysis revealed considerable regional heterogeneity in the number of detected features: Heilongjiang (3110), Jilin (3721), Liaoning (2182), Tianjin (1858), Hebei (2567), Henan (3658), Shandong (4592), and Guangdong (4701) (Figure S3). Significant positive correlations between soil organic carbon content (OC%) and feature number were identified in Guangdong, Heilongjiang, Liaoning, and Shandong Provinces (Figure S4), indicating hydrophobicity-mediated sorption mechanisms. Conversely, Hebei, Henan, Tianjin, and Jilin exhibited no significant OC%–feature relationships. This spatial divergence likely stemmed from differential anthropogenic inputs, variable pollution source intensities, regional microbial community variations, and site-specific environmental conditions [53,54].

#### 3.2.2. Spatial Distribution of Micropollutant Proportions

**Figure 2:**
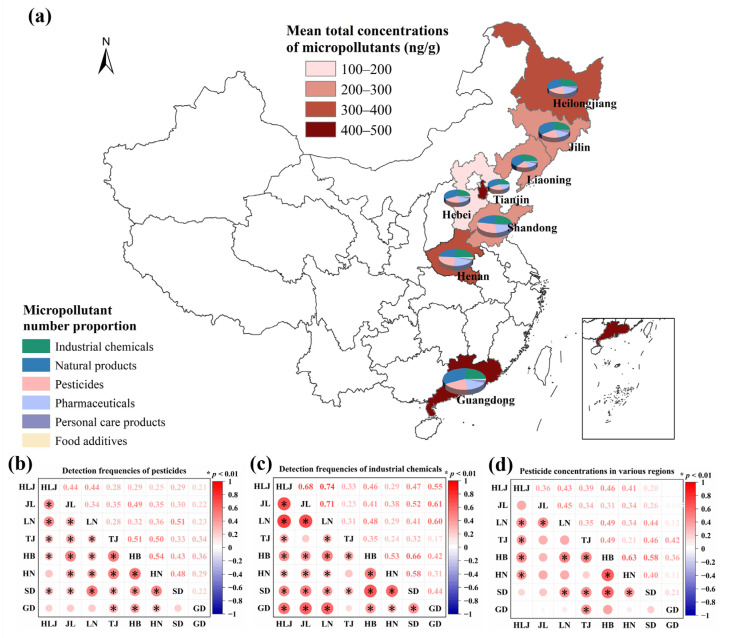
Regional variations in micropollutant characteristics across eight regions in China. (**a**) Spatial distribution of mean total micropollutant concentrations and categorical composition; Spearman’s correlations of regional patterns in (**b**) pesticide detection frequency, (**c**) industrial chemical detection frequency, and (**d**) pesticide concentration.

The compositional distribution of six micropollutant categories demonstrated distinct regional patterns (Figure 2a). Liaoning and Heilongjiang Provinces exhibited elevated proportions of industrial chemicals, consistent with their historical role as northeastern China’s heavy industry hubs [55]. Shandong demonstrated the highest proportion of pesticides, aligning with its intensive vegetable cultivation and documented high pesticide application rates [56]. Personal care products and food additives demonstrated consistently low proportions across all regions, indicating incidental anthropogenic inputs comparable to literature reports [57]. Spearman correlation analyses of DFs provided further spatial insights (Figure 2b). A significant positive correlation (*r* = 0.54, *p* < 0.01) for pesticide DFs between the adjacent provinces, Henan and Hebei suggested shared agricultural practices—including wheat-maize rotations, overlapping pesticide usage due to common suppliers, and similar pest management strategies [58]. Comparable climatic conditions likely reinforced this correlation by consistently influencing pesticide degradation, volatilization, or rainfall-mediated transport processes [59]. Industrial chemicals exhibited pronounced spatial correlations, with northeastern provinces (Heilongjiang, Jilin, and Liaoning) showing high inter-provincial DF correlations (*r* = 0.68–0.74, *p* < 0.01, Figure 2c). This aligned with their historical concentration of heavy industries (e.g., chemical manufacturing, metallurgy, and machinery production), where legacy emissions may contribute to persistent compound accumulation via atmospheric deposition or surface runoff [60]. Enhanced retention was further facilitated by the relatively high sorption capacity of organic-rich black soils in these provinces (OC% = 1.9–8.7%, mean: 4.26%, Table S1). Significant DF correlations for industrial chemicals also existed among northern provinces (Hebei, Henan, and Shandong; r = 0.53–0.66, *p* < 0.01, Figure 2c), potentially attributable to geographic proximity, integrated industrial ecosystems, and shared reliance on traditional heavy industries employing similar chemical inputs. Notably, the DFs of industrial chemicals in Guangdong correlated significantly with those in Jilin, Heilongjiang, and Liaoning (r = 0.55–0.61, *p* < 0.01, Figure 2c). While northeastern provinces reflected historical accumulation from past industrial activity, Guangdong–currently China’s largest industrial province by output (https://www.stats.gov.cn/sj/ndsj/2021/indexch.htm, accessed on 26 July 2025)–likely exhibited contamination primarily driven by contemporary emissions from modern manufacturing. This suggests distinct temporal pathways (historical persistence versus recent emissions) can yield analogous spatial distribution patterns for certain industrial chemicals.

#### 3.2.3. Spatial Distribution of Micropollutant Concentrations

The mean total concentrations of 50 quantified micropollutants ranged from 106 to 445 ng/g across different regions (Figure 2a and Table S5), revealing a pronounced latitudinal contamination gradient. Crucially, pesticides constituted 83.6–95.8% of the micropollutant concentrations based on our analytical data, establishing them as the primary drivers of spatial contamination patterns. Guangdong exhibited the highest mean concentrations of micropollutants, whereas Hebei showed the lowest, reflecting regional differences in cropping systems, climatic conditions, and agrochemical management. In Guangdong, intensive agrochemical applications in perennial orchard cultivation combined with subtropical pest pressures drove significant accumulation of micropollutants [61]. Although Tianjin has a temperate climate, all sampling sites were situated in vegetable soils. The high planting densities associated with this land use led to persistently high residual levels of pollutants [62]. Conversely, Jilin, Liaoning, and Hebei showed lower micropollutant concentrations, which were partially due to corn-soybean cultivation systems with reduced pesticide inputs [63]. Spearman correlation analysis of pesticide concentrations (Figure 2a) revealed significant positive correlations between Hebei-Henan (r = 0.63, *p* < 0.01) and Hebei-Shandong (r = 0.58, *p* < 0.01). The weak inter-regional correlations between Guangdong and other regions highlighted how distinctive regional signatures—such as cropping systems, climatic conditions, and agrochemical management—governed micropollutant profiles.

**Figure 3:**
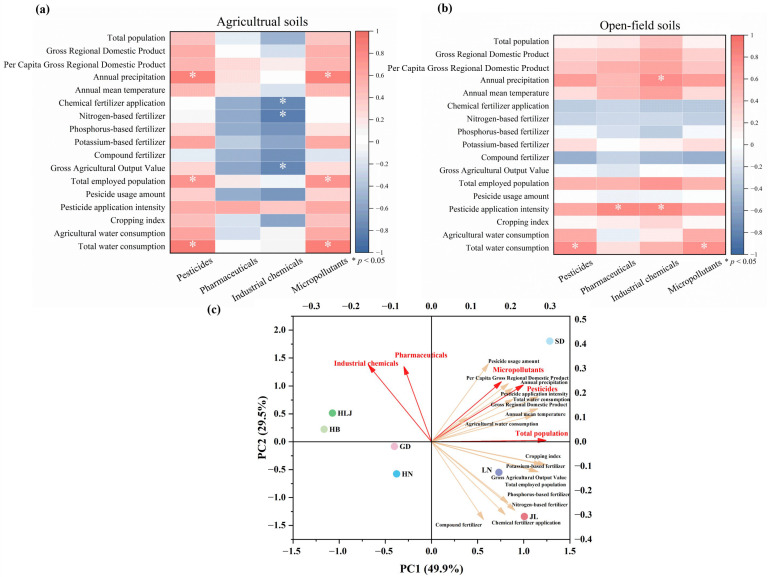
Correlations between micropollutant concentrations in each region and environmental and socioeconomic parameters: (**a**) agricultural soils, (**b**) open-field soils. (**c**) PCA biplot of micropollutants and environmental/socioeconomic variables, highlighting regional differences in dominant drivers.

To explore potential large-scale drivers of micropollutant distribution, we conducted a preliminary correlation analysis between regional micropollutant concentrations and environmental and socioeconomic parameters derived from provincial statistical yearbooks (Figure 3 and Table S7) [21], excluding Tianjin to ensure provincial-scale analytical consistency. It was critical to emphasize that, given the limited number of sampling sites per region, these correlations were not intended to establish definitive causal relationships. Instead, they served to identify suggestive patterns and generate hypotheses regarding the synergistic effects of climate, agricultural practices, and socioeconomic factors on a national scale. The findings warranted validation through future studies with more intensive, stratified sampling designs. Our analysis revealed significant positive correlations between micropollutant/pesticide concentrations and annual precipitation (r = 0.88, *p* < 0.05, Figure 3a), alongside moderate associations with annual mean temperature (r = 0.50, *p* > 0.05, Figure 3a). Paradoxically, while precipitation typically enhances pollutant migration and loss [64], it was positively correlated with soil residue levels in this study. We proposed that precipitation-driven agricultural activities—such as increased pesticide application—override the dilution and leaching effects of rainfall. This was particularly evident under conditions of high precipitation and temperature, where more frequent use led to net accumulation in soils [61]. Further analysis demonstrated positive correlations between micropollutant/pesticide concentrations and total water consumption (*r* = 0.77–0.78, *p* < 0.05) and agricultural water consumption (*r* = 0.59–0.62, *p* > 0.05), implicating irrigation practices in micropollutant retention. Moderate correlations were observed between micropollutant/pesticide concentrations and regional pesticide application intensity (*r* = 0.62–0.64, *p* > 0.05), indicating direct chemical input as an accumulation driver, while moderate associations with cropping index (*r* = 0.44–0.48, *p* > 0.05) suggested that frequent planting cycles intensified micropollutant loading. Socioeconomic linkages emerged through significant positive correlations with total employed population (*r* = 0.75, *p* < 0.05), alongside moderate correlations for Gross Regional Domestic Product (*r* = 0.58–0.59, *p* > 0.05) and Per Capita Gross Regional Domestic Product (*r* = 0.52–0.53, *p* > 0.05), collectively connecting micropollutant persistence to anthropogenic activity. Pharmaceuticals exhibited no significant correlations with the investigated parameters. Industrial chemical concentration showed a negative correlation with Gross Agricultural Output Value (*r* = 0.79, *p* < 0.05, Figure 3a), indicating that provinces with higher agricultural output feature large-scale farming practices but fewer industrial pollution sources. Notably, industrial chemical concentrations in open-field soils correlated significantly with annual precipitation (*r* = 0.78, *p* < 0.05, Figure 3b), implicating atmospheric deposition as a significant accumulation pathway. Collectively, these findings established that micropollutant spatial distribution was co-regulated by climatic variables, agricultural management factors, and socioeconomic drivers.

The Principal Component Analysis (PCA) results (Figure 3c) indicated that Heilongjiang, Hebei, Henan, and Guangdong clustered near the central region of the plot, away from the main loading vectors, suggesting that micropollutant levels in these provinces are influenced by multiple environmental and socioeconomic factors, with no single dominant driver [65]. Shandong was located in the positive quadrant of both PC1 and PC2, closely associated with pesticide application, micropollutant concentrations, and demographic-economic indicators, reflecting a clear dual influence of agricultural input and socioeconomic activity. Liaoning and Jilin aligned with the vectors for fertilizer application and cropping index, representing typical fertilizer-driven regions. Overall, the PCA regional patterns were consistent with the previous correlation analysis, further confirming that provincial micropollutant distributions are jointly regulated by agricultural intensity, climate, and socioeconomic factors, while also validating the observed similarities in micropollutant detection frequencies and pesticide concentrations between Henan and Hebei.

### 3.3. Distribution of Micropollutants Across Greenhouse and Open-Field Soils

**Figure 4:**
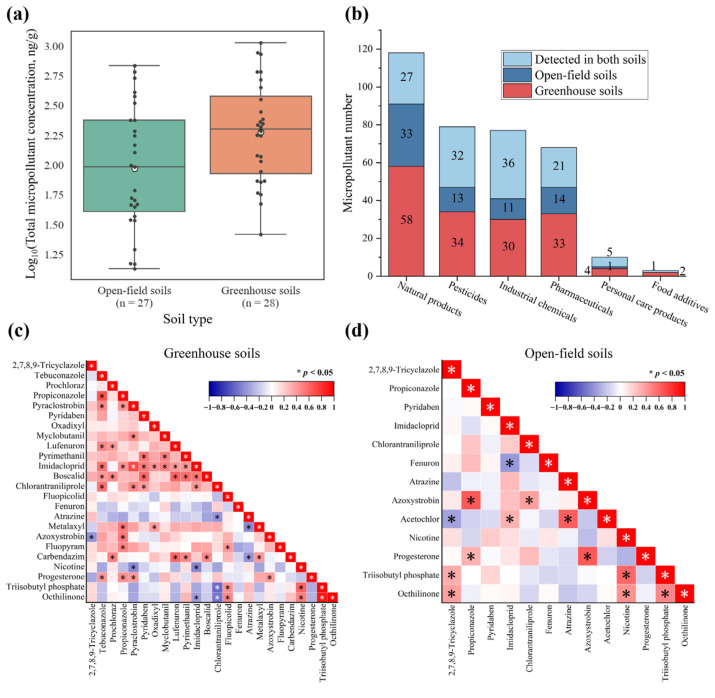
(**a**) Numbers of micropollutants by category, differentiated by detection in greenhouse soils, open-field soils, and both soils. (**b**) Comparison of log-transformed total micropollutant concentrations between greenhouse soils (*n* = 28) and open-field soils (*n* = 27). (**c**) Correlation among micropollutant concentrations in greenhouse soils. (**d**) Correlation among micropollutant concentrations in open-field soils.

Nearly equivalent numbers of greenhouse soil (*n* = 28) and open-field soil (*n* = 27) samples were collected across seven regions (excluding Guangdong). As illustrated in Figure S5, greenhouse soils exhibited higher feature numbers compared to open-field soils in most regions. Furthermore, greenhouse soils contained greater quantities of micropollutants across all compound categories relative to open-field systems (Figure 4b). The concentrations of 50 quantified micropollutants were higher in greenhouse soils than in open-field soils (Figure 4a). This enhanced accumulation in the greenhouse environments likely stemmed from multiple factors. The extensive plastic film usage contributed to microplastic accumulation in the soils (e.g., plasticizer benzyl butyl phthalate detected at sites JL-6, SD-7, and HB-3). Greenhouse structures also impeded natural attenuation by blocking phototransformation-active wavelengths [66]. Furthermore, pesticide applications and wastewater irrigation constitute primary sources of soil micropollutants. Greenhouse systems exhibited particularly intensive cultivation regimes where heightened pesticide application frequency and diversity [67], combined with irrigation-mediated pollutant inputs, collectively elevated micropollutant loads. Although elevated temperatures and humidity in greenhouses may accelerate certain micropollutant transformations [68], net accumulation remained higher due to the combined effects of intensive chemical inputs, restricted transport pathways, and limited environmental turnover. Conversely, open-field soils experience enhanced attenuation through rainfall-driven leaching, surface runoff, phototransformation, and microbial degradation [69]. Collectively, greenhouse soils demonstrated higher micropollutant diversity and residue levels than open-field soils, highlighting the need for targeted management strategies. A strong positive correlation (r = 0.76, *p* < 0.001) was found in the DFs of 498 micropollutants between greenhouse and open-field soils (Figure S6), indicating shared pollution sources. However, the correlation for concentrations between these soil types was significantly weaker (r = 0.28, *p* < 0.05, Figure S7). This differential correlation pattern further indicated distinct transport and transformation processes for micropollutants following their entry into different agricultural systems.

#### 3.3.1. Co-Occurrence of Micropollutants in the Greenhouse and Open-Field Soils

The proportion of overlapping features between greenhouse and open-field soils ranged from 23.1% to 40.3% of total features per region (Figure S5). When aggregating all regional samples, co-occurrence rates differed markedly among micropollutant categories (Figure 4a). High co-occurrence rates were observed for personal care products (50.0%), industrial chemicals (46.8%), and pesticides (40.5%), suggesting consistent introduction via agricultural and environmental pathways. For instance, certain pesticides are ubiquitously applied across cultivation systems for weed and pest control [70]. Industrial chemicals and personal care products likely entered soils through indirect routes such as sewage irrigation [71], organic fertilizers [72], or atmospheric deposition [73]. Despite semi-enclosed conditions, greenhouse soils received these micropollutants through intensive fertilization and irrigation. Moreover, these pollutants persist in the environment due to their low biodegradability, strong hydrophobicity, or high photostability, leading to their accumulation across various soil types [74]. Representative co-occurring compounds included chlorophene (disinfectant in personal care products), 1,3-diphenylguanidine (rubber processing additive), and 2,7,8,9-tricyclazole (vegetable cultivation fungicide). Their consistent detection underscored the need to investigate environmental fate and the long-term ecological risks.

Conversely, lower rates of co-occurrence between greenhouse and open-field soils were observed for natural products (22.9%), pharmaceuticals (30.9%), and food additives (33.3%), potentially due to divergent sources and transformation kinetics. Greenhouse cultivation prioritizes fast-maturing cultivars, while open-field systems select stress-resistant varieties, leading to differing root exudate profiles [75]. Distinct soil microbiomes and environmental conditions further influenced microbial metabolic activities [76], collectively regulating natural product compositions. Greenhouse systems frequently employ high-intensity inputs like livestock manure, organic liquid fertilizers, or seedling substrates, whereas open-field systems utilize diverse organic amendments, yielding more varied pharmaceutical profiles [77]. Rainfall-driven runoff in open fields additionally mobilized and diluted pharmaceutical-derived micropollutants. The inherent instability of some natural products and pharmaceuticals results in transformation rates that vary across environments [78]. Although food additives may enter soils via wastewater irrigation or sewage sludge application, their lower co-occurrence rates likely reflected reduced emission intensities and usage frequencies, coupled with high water solubility and high transformation rates that limited environmental persistence [79]. Unlike agricultural chemicals introduced via direct application, food additives predominantly entered soils through incidental anthropogenic pathways. Consequently, sporadic inputs constrained consistent detection in both greenhouse and open-field soils.

#### 3.3.2. Correlation Analysis of Micropollutants in Greenhouse and Open-Field Soils

Spearman correlation analyses revealed distinct pairwise relationships among micropollutant concentrations between greenhouse and open-field soils (Figure 4c,d). Greenhouse soils exhibited 45 significant positive correlations (r = 0.38–0.90, *p* < 0.05) and 9 negative correlations (r = 0.38–0.58, *p* < 0.05), whereas open-field soils showed fewer significant associations: 11 positive correlations (r = 0.31–0.65, *p* < 0.05) and 2 negative correlations (r = −0.39 to −0.34, *p* < 0.05). Representative associations included the positive correlation between the insecticides imidacloprid and pyridaben in greenhouse soils (r = 0.50, *p* < 0.05), herbicides acetochlor and atrazine in open-field soils (r = 0.62, *p* < 0.05), and fungicides azoxystrobin and propiconazole across both soil systems (r = 0.53–0.64, *p* < 0.05). These patterns likely reflected co-application practices in commercial pesticide formulations or integrated pest management strategies [80]. Compounds with similar physicochemical properties (e.g., triazole fungicides propiconazole and tebuconazole) and environmental stability may also demonstrate analogous behavior [81]. The reduced correlation frequency in open-field soils may originate from greater environmental variability affecting micropollutant fate. Notably, positive correlations between industrial chemicals (triisobutyl phosphate and octhilinone) and pharmaceutical nicotine in both systems suggested a shared pollution source, potentially from irrigation water [82].

### 3.4. Distribution of Micropollutants Across Cropping Types

**Figure 5:**
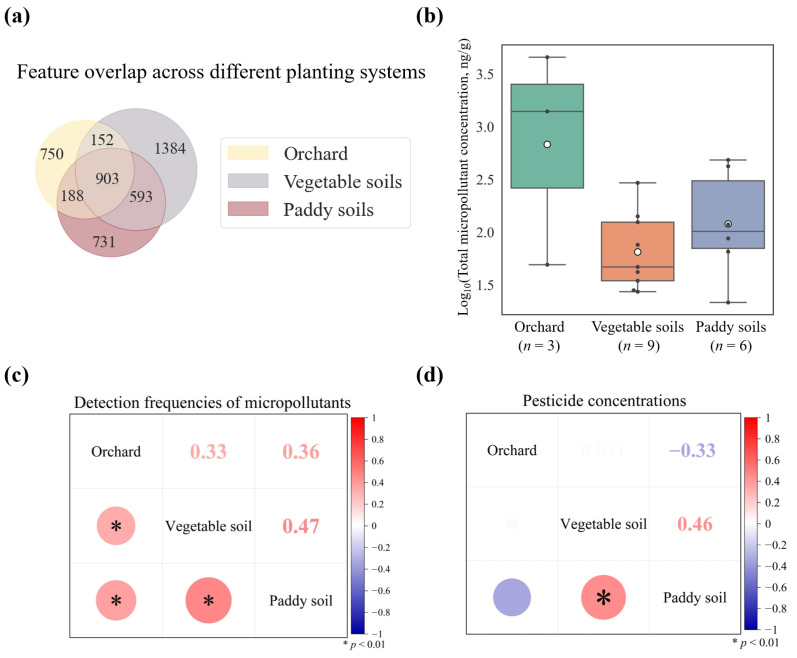
Characteristic features and differential micropollutant accumulation across agricultural soil systems in Guangdong Province. (**a**) Venn diagram of unique and shared features in orchard, vegetable, and paddy soils. (**b**) Micropollutant concentrations across agricultural systems. (**c**) Correlation matrix for micropollutant detection frequencies. (**d**) Correlation matrix for micropollutant concentrations.

To evaluate the impact of different crops on soil pollutants, we analyzed samples from three crop types in Guangdong Province: orchard soils (*n* = 3), paddy soils (*n* = 6), and vegetable soils (*n* = 9). Distinct pollutant profiles were observed across these soils. Although vegetable soils contained the highest total number of unique features (3032, Figure 5a), the average number of features per site was highest in orchard soils (664), followed by paddy soils (403) and vegetable soils (337). This pattern was consistent with measured concentrations: micropollutant levels were highest in orchard soils, substantially exceeding those in paddy and vegetable soils (Figure 5b). Pairwise correlation analysis showed that the detection frequencies of micropollutants were significantly correlated among orchard, vegetable, and paddy soils (Figure 5c). Although all three soils are located within a humid subtropical climate, the elevated accumulation of pollutants in orchard soils was likely attributable to the infrequent cultivation practices employed for perennial fruit trees, coupled with single-dose applications of high-concentration pesticides for pests and disease control [83]. In contrast, regular irrigation and tillage in rice paddies, along with short-cycle, high-frequency tillage in vegetable systems, enhanced micropollutant mobility and reduced accumulation [84]. Furthermore, elevated residue levels of certain orchard pesticides (chlorpyrifos, propiconazole, triazole, and fluoxastrobin) contributed significantly to the overall micropollutant burden; for instance, chlorpyrifos reached a peak concentration of 2786 ng/g.

Feature overlap analysis demonstrated substantial co-occurrence differences: orchard-vegetable soils shared 1055 features, orchard-paddy soils 1091, and vegetable-paddy soils 1496 (Figure 5a). Vegetable-paddy systems exhibited both the highest feature overlap (*n* = 1496) and significantly correlated pesticide concentrations (r = 0.46, *p* < 0.01, Figure 5c), potentially attributable to agronomic similarities including short growth cycles and rotational practices [85]. Interestingly, despite substantial feature overlap between orchard and paddy soils (*n* = 1091), their pesticide concentrations showed a negative correlation (r = −0.33, Figure 5d). This divergence likely stemmed from differential management: rice cultivation involves ploughing and flooding that promote pesticide dilution, while orchards experience limited tillage, enabling soil accumulation [17]. Regular paddy field drainage further removed some water-soluble pesticides [86]. Collectively, these findings demonstrated that micropollutant accumulation was intrinsically linked to crop-specific management practices, land-use intensity, and environmental conditions governing micropollutant behavior.

### 3.5. Environmental Risk Assessment

**Figure 6:**
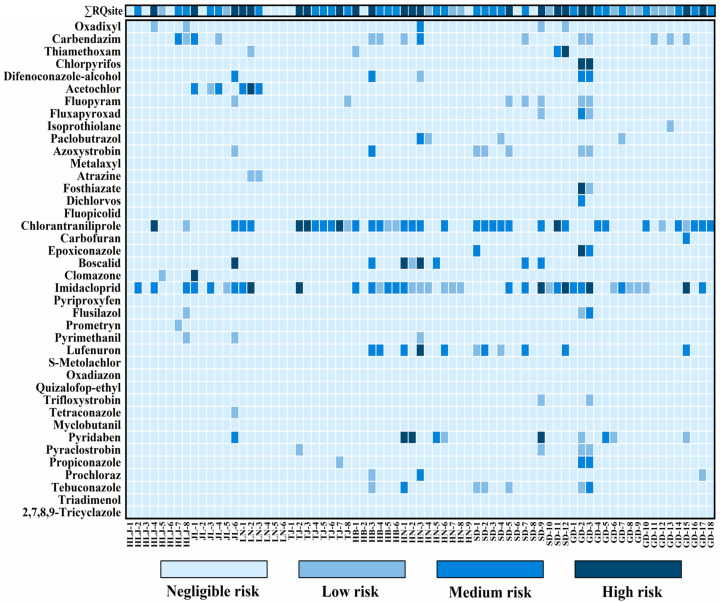
Ecological risk profiles of soil micropollutants: Risk classification at 73 sampling sites with cumulative risks (Σ*RQ* _site_).

Given limited toxicological data, this study assessed *RQ*s for 40 pesticides (Table S8), with site-specific Σ*RQ*_site_ values calculated by summing individual *RQ*s per sampling location. Our analysis revealed: 12 sites (16.4%) at negligible risk, 12 (16.4%) at low risk, 28 (38.4%) at moderate risk, and 21 (28.8%) at high risk across all regions (Figure 6). This high-risk proportion aligned with reports from the Three Gorges Reservoir (31.3%) [27]. Eleven pesticides exhibited high risks (*RQ* ≥ 1) at one or more site: pyridaben, lufenuron, imidacloprid, clomazone, boscalid, epoxiconazole, chlorantraniliprole, fosthiazate, acetochlor, chlorpyrifos, and thiamethoxam. These compounds warrant long-term monitoring and application restrictions. Chlorpyrifos posed the highest risk in Guangdong (*RQ* = 2322) due to its extremely low *PNEC*_mss_ (1.2 ng/g) and high measured concentration (2787 ng/g). The environmental persistence of chlorpyrifos and imidacloprid, coupled with application rates exceeding recommended doses, contributed significantly to ecological risks [87]. These findings suggested that current pesticide regulations require stricter limits for highly toxic compounds with elevated residue levels. While no pronounced regional differences emerged in risk distribution, medium-to-high risk sites (Σ*RQ*_site_ ≥ 0.1) constituted ≥50% of sites in all regions. Particularly high proportions occurred in Tianjin, Hebei, and Shandong (75.0–87.5%). Greenhouse soils exhibited higher risk (Σ*RQ*_site_) proportions: 35.7% high-risk, 32.1% moderate-risk, 10.8% low-risk, and 21.4% negligible-risk sites. Open-field soils showed lower high-risk incidence (24.4%) but higher moderate-risk (42.2%), with 20.1% low-risk and 13.3% negligible-risk sites. Pesticide mixtures exert synergistic ecological effects even at trace concentrations. Primary risk drivers diverged between systems: greenhouse high-risk sites (*n* = 10) were dominated by boscalid, chlorantraniliprole, imidacloprid, pyridaben, and thiamethoxam, while open-field sites relied on chlorantraniliprole, imidacloprid, clomazone, acetochlor, and chlorpyrifos (Figure S8). Imidacloprid (2.6–76.9%) and chlorantraniliprole (3.1–99.9%) substantially impacted >80% of high-risk sites, warranting prioritized regulation. Other pesticides contributed minimally to cumulative risks. Overall, while seasonal climate and agricultural practices obscured clear regional *RQ* patterns, greenhouse soils consistently exhibited greater contamination severity than open-field systems. In summary, among the identified high-risk pesticides, a small subset of pesticides—particularly imidacloprid, chlorantraniliprole, chlorpyrifos, and pyridaben—emerged as the main drivers of ecological risk. Their high persistence and low PNEC values led to disproportionately high RQ contributions even at trace concentrations. These results indicate that future monitoring and regulatory efforts should focus on these high-risk pesticides, especially in greenhouse systems, and that mitigation strategies such as crop rotation and enhanced biodegradation could help reduce ecological impacts and support sustainable agricultural management.

## 4. Conclusions and Limitations

This study explored the occurrence and distribution of organic micropollutants in agricultural soils across major food-producing regions in China. Through non-targeted screening, we identified 498 micropollutants, spanning pesticides, pharmaceuticals, industrial chemicals, personal care products, natural products, and food additives. This comprehensive inventory included banned pesticides, regulated priority substances, and emerging contaminants, demonstrating the feasibility and value of non-targeted screening for large-scale soil monitoring. Spatial analysis revealed significant regional patterns, with pesticide DFs showing strong correlations between Henan and Hebei Provinces, likely reflecting shared cropping systems and broadly similar pesticide use practices. Micropollutant concentrations were significantly associated with precipitation, water consumption, employed population, and agricultural output, highlighting the interplay of climatic, agricultural, and socioeconomic factors. A notable finding was the generally higher quantity, diversity, and concentration of micropollutants in greenhouse soils compared to open-field soils. This disparity was attributed to intensive agrochemical applications in greenhouse soils and the containment effect of plastic sheeting, which hindered the environmental migration and degradation of micropollutants. Higher co-occurrence rates of personal care products, industrial chemicals, and pesticides in both greenhouse and open-field soils suggested common input pathways and environmental persistence. In contrast, the lower co-occurrence of natural products and pharmaceuticals resulted from crop-specific root exudates and rainfall-induced leaching of hydrophilic substances. Among cropping systems, orchard soils exhibited the highest micropollutant concentrations, followed by paddy soils and vegetable soils. This pattern was linked to high-concentration pesticide applications in perennial fruit trees, whereas regular tillage in paddy soils and high-frequency tillage in vegetable soils enhanced micropollutant mobility and reduced accumulation. Risk quotients indicated moderate-to-high ecological risks at >50% of sites, with greenhouse systems consistently demonstrating higher risks than open fields despite homogeneous regional risk distribution. These findings have important implications for soil health, food safety, and human exposure. Elevated levels of persistent pesticides and industrial chemicals can disrupt soil microbial communities, nutrient cycling, and ecosystem functions, and high-risk compounds in greenhouse and orchard soils pose tangible threats to crop safety and dietary human exposure. Moreover, micropollutants can leach into groundwater or be transported via surface runoff, increasing exposure through drinking water and environmental contact, highlighting the necessity for integrated soil management and targeted mitigation strategies.

Overall, this study highlights greenhouse and orchard systems as priority targets for pollution control and risk mitigation in China’s major food-producing regions. The findings underscore the urgent need for region-specific and cropping-system-oriented management strategies, including optimized pesticide application, improved regulation of greenhouse agrochemical use and plastic film management, and routine monitoring of high-risk areas, to reduce micropollutant inputs, limit their environmental mobility, and safeguard soil health, food safety, and human well-being.

While this study provided broad chemical coverage and new insights into cultivation-specific pollution patterns, several limitations should be acknowledged. First, the sample size in some central and northeastern Chinese regions was limited due to their similar climatic and agricultural conditions, which may affect the detection of region-specific compounds and reduce data representativeness at the provincial scale. Future research should focus on increasing sampling density within key regions and implementing multi-seasonal monitoring to improve spatial and temporal resolution. Second, although a wide-spectrum LC-HRMS (ESI) workflow was employed, certain pollutant classes inherently fall outside its analytical window. Hydrophobic and thermally stable compounds, such as polycyclic aromatic hydrocarbons (PAHs) and legacy organochlorine pesticides (e.g., DDTs), cannot be efficiently ionized in the ESI source and are better detected using GC-MS; thus, they were not captured in this study. Similarly, highly polar substances such as per- and polyfluoroalkyl substances (PFASs) exhibit poor ionization efficiency under the applied LC-ESI conditions, and the lack of WAX-based enrichment further limited their detection. These compound classes were therefore beyond the scope of the present analysis and should be incorporated in future studies using complementary GC-MS or LC-MS/MS methods optimized for these chemicals.

## Data Availability

The original contributions presented in this study are included in the article/Supplementary Material. Further inquiries can be directed to the corresponding author.

## Supplementary Materials

The following supporting information can be downloaded at https://www.mdpi.com/article/10.3390/toxics14010025/s1, Table S1: Detailed information on sampling sites across different regions; Text S1: Detailed soil extraction and purification protocols; Table S2: Recoveries, method detection limits (MDLs), and method quantification limits (MQLs) of micropollutants; Text S2: Instrumental parameters; Table S3: Predicted-no-effect concentrations (*PNEC*mss) for micropollutants in the soils; Figure S1: Representative spectra for micropollutants with confidence level 1 and 2; Table S4: Comprehensive data on micropollutants; Table S5: Mean total concentration of micropollutants in each province and municipality; Figure S2: Subcategories of pesticides, pharmaceuticals, and industrial chemicals; Table S6: Comparison of reported pesticide concentration ranges in agricultural soils from representative studies worldwide; Figure S3: Total number of features detected in each region; Figure S4: Correlation between the soil organic carbon content and the total number of features in each sampling site; Table S7: Provincial environmental and socioeconomic data for preliminary correlation with micropollutant concentrations; Figure S5: Distribution of regional features in greenhouse and open-field soils. Figure S6: Pearson correlation of detection frequencies (DFs) of micropollutants (n = 498) in greenhouse and open-field soils; Figure S7: Correlation between micropollutant concentrations between greenhouse soils and open-field soils; Table S8: Risk quotients (*RQ*s) for micropollutants in 73 soil samples; Figure S8: Relative contribution of individual pesticides to the overall risk at 21 high-risk sites (Σ*RQ*_site_ ≥ 1) [10,17,38,39,88,89,90,91,92].

## Abbreviations

The following abbreviations are used in this manuscript:

HRMS	High-resolution mass spectrometry
ISTD	Isotopically labeled internal standards
HPLC	High-performance liquid chromatograph
QTOF-MS	Quadrupole time-of-flight mass spectrometer
ESI+	Electrospray ionization positive
ESI-	Electrospray ionization negative
TQ-MS	Triple quadrupole mass spectrometer
RT	Retention time
MDLs	Method detection limits
MQLs	Method quantification limits
S/N	Signal-to-noise (S/N) ratios
RQ	Risk quotient
MEC_soil_	Measured environmental concentration in soil
PNEC_mss_	Predicted no-effect concentration for micropollutants in soils
NOEC	No-observed-effect concentration
LC_50_	Median lethal concentration
AF	Assessment factor
ND	Not detected
DF	Detection frequency
IARC	International Agency for Research on Cancer
OC%	Organic carbon content
