# Supplementary material for: Non-Targeted and Targeted Analysis of Organic Micropollutants in Agricultural Soils Across China: Occurrence and Risk Evaluation
Source: Toxics. 2025 Dec 25;14(1):25. doi: 10.3390/toxics14010025 (PMC12845987; doi:10.3390/toxics14010025)
Supplement: Supplementary file 1 [file toxics-14-00025-s001.zip › toxics-4044453-supplementary.pdf]

Supplementary information

Non-targeted and targeted analysis of organic micropollutants in agricultural soils across China: Occurrence and risk evaluation

Table S1. Detailed information on sampling sites across different regions (Please see more details in uploaded excel file).

Text S1. Detailed soil extraction and purification protocols

Samples were spiked with 6 mL of methanol, vortexed (30 s), ultrasonicated (20 min), and centrifuged (4000 rpm, 10 min). The supernatants were collected and transferred to brown glass bottles. The extraction procedure was repeated three times, and the combined extracts were diluted to a volume of 600 mL with water. The diluted extracts were loaded onto solid-phase extraction (SPE) cartridges (Oasis PriME HLB, 6 cc, 500 mg), which were preconditioned with methanol (10 mL) and water (5 mL). The SPE system was operated under vacuum at 4–5 mL/min until all the liquid had passed through the SPE cartridges. Then, the analytes were eluted with 6 mL of methanol and concentrated to 0.5 mL under a gentle nitrogen stream (purity ≥ 99.999%) and were reconstituted with a methanol–water mixture prior to instrumental analysis.

Text S2. Instrumental parameters

The mobile phase for ESI+ mode consisted of water containing 5 mmol/L ammonium acetate and 0.1% formic acid (A) and methanol with 0.1% formic acid (B), delivered at a constant flow rate of 0.4 mL/min. For ESI- mode, the mobile phase comprised 5 mmol/L ammonium acetate in water (A) and pure methanol (B). Both ionization modes employed identical chromatographic gradients: initial conditions of 5% B were held for 1 min, followed by a ramp to 50% B over the next 3 min; the 50% composition was maintained until 17 min; subsequently, a gradient increased B to 100% at 20 min where it was held constant; finally, at 20.1 min the composition was immediately reduced to 5% B and maintained until 22.5 min.

Mass spectrometry data acquisition was performed in ESI + and ESI- modes using both Auto MS/MS and All-Ions Fragmentation (AIF). Spectral information was acquired across the *m*/*z* range 50-1000 Da. AIF analysis employed collision energies of 0 eV and 30 eV at a scan rate of 5 Hz, ensuring sufficient data points for accurate production of chromatographic peaks. Auto MS/MS analysis captured fragmentation data for the seven secondary fragments with the highest corresponding intensities, using collision energies and scan rates identical to AIF methods. For quantitative analysis, the mobile phase consisted of water (A) containing 5 mmol/L ammonium acetate and 0.1% formic acid and methanol with 0.1% formic acid (B), with an identical

gradient elution program to that used for HPLC-QTOF-MS separation.

Table S2 Recoveries, method detection limits (MDLs), and method quantification limits (MQLs) of micropollutants (Please see more details in uploaded excel file).

Table S3 Predicted-no-effect concentrations ($PNEC_{mss}$) for micropollutants in the soils (Please see more details in uploaded excel file).

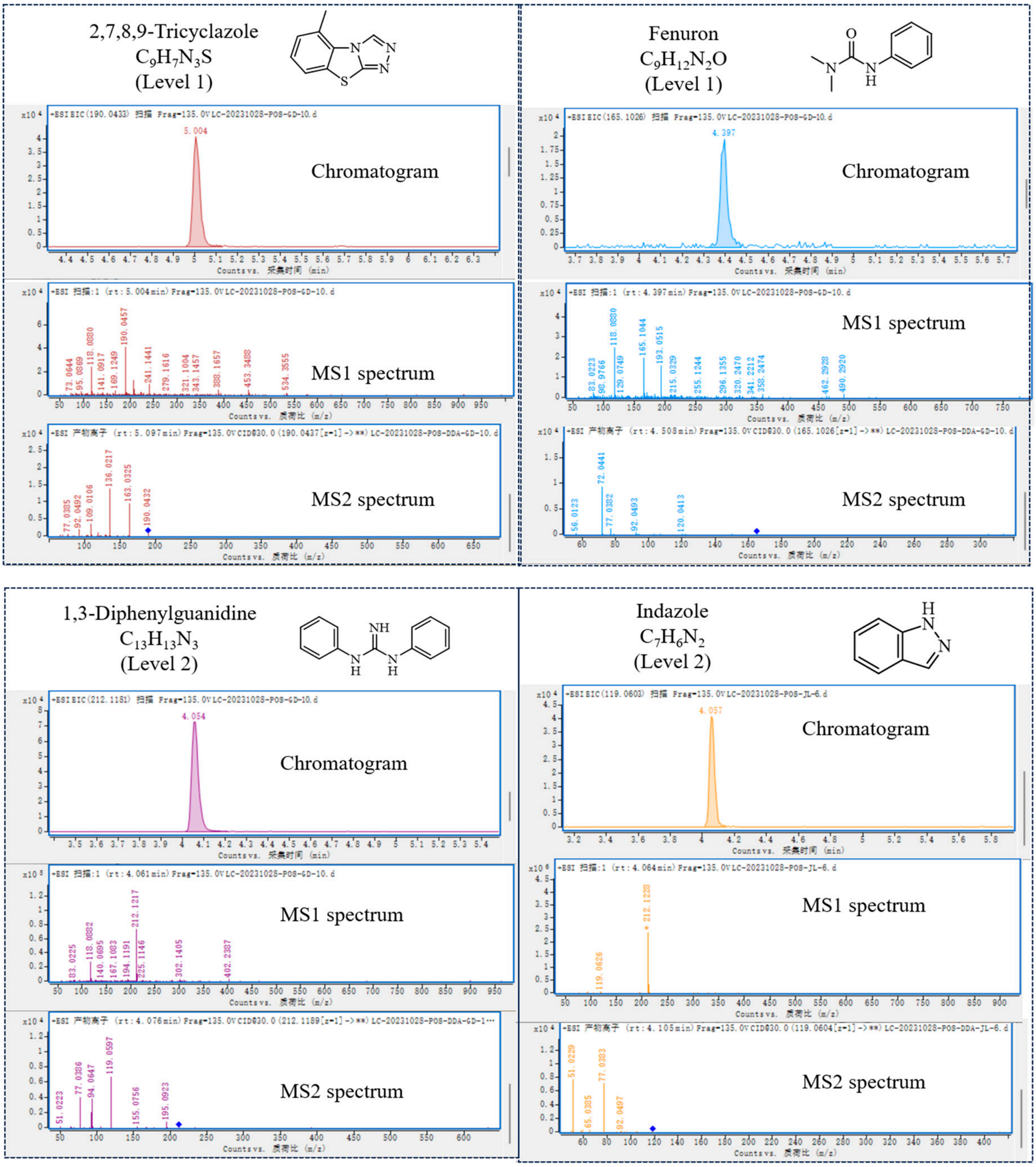

Figure S1 Representative spectra for micropollutants with confidence level 1 and 2.

Table S4 Comprehensive data on micropollutants (Please see more details in uploaded excel file).

Table S5 Mean total concentration of micropollutants in each province and municipality (Please see more details in uploaded excel file).

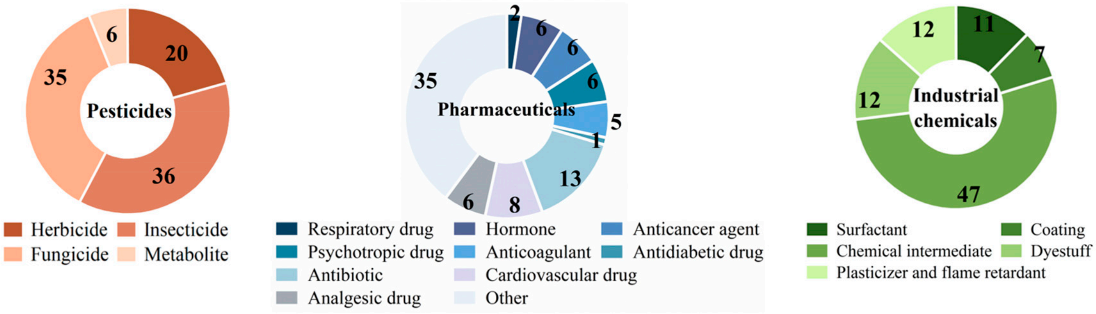

Figure S2 Subcategories of pesticides, pharmaceuticals, and industrial chemicals.

Table S6. Comparison of reported pesticide concentration ranges in agricultural soils from representative studies worldwide.

Study region	Total cumulative concentration (ng/g)	Median/mean (ng/g)	Soil type	Reference
France	0.08–1274	/	Arable lands, orchards	(Froger et al., 2023)
China	1.05–327	55.8	Peach orchard soils	(Li et al., 2023)
Europe	ND–2870	1990	Agricultural top soils	(Silva et al., 2019)
Czech Republic	2.0–269	/	Arable soils	(Hvezdová et al., 2018)
Switzerland	ND–1170	90	Vegetable soils	(Riedo et al., 2021)
Nepal	1.0–251	16	Vegetable soils	(Bhandari et al., 2020)
China (Dongting Lake Polder)	5.2–663	140	Agricultural soils	(Guo et al., 2025)
China (Heilongjiang)	189–845	/	Agricultural soils	(Wang et al., 2024)
Finland	ND–3043	534	Arable soils	(Hagner et al., 2024)

[a] ND: not detected.

[b] "/" indicates data not reported in the original study.

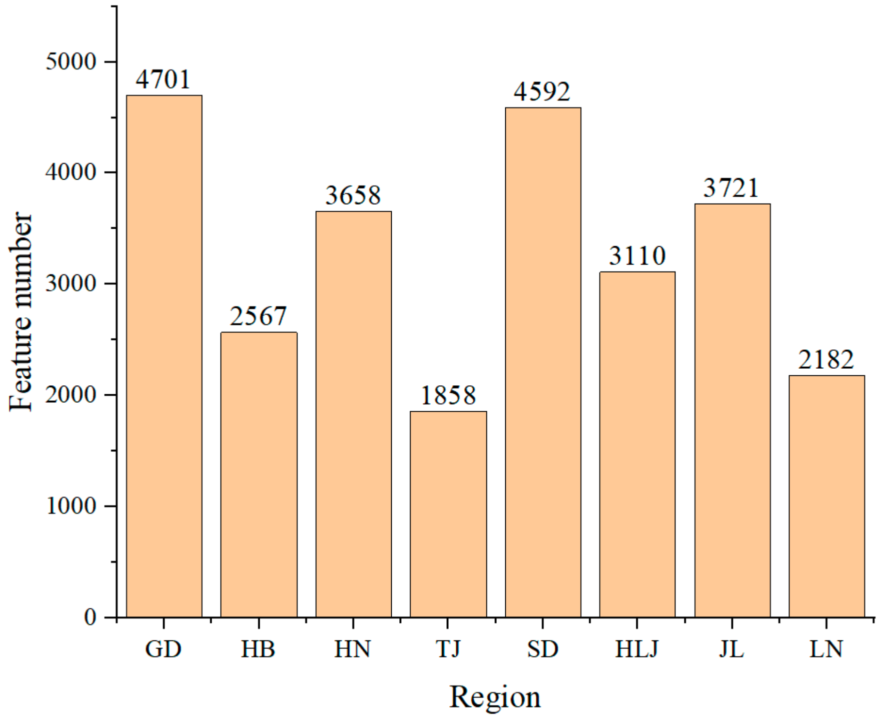

Figure S3 Total number of features detected in each region.

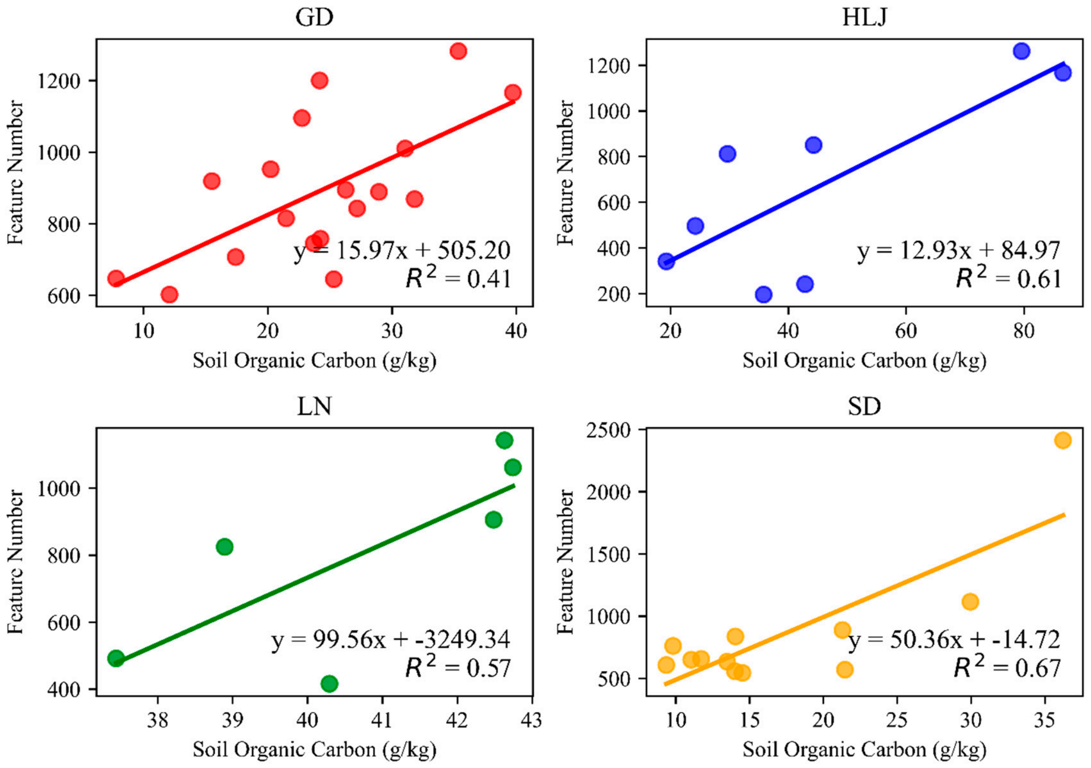

Figure S4. Correlation between the soil organic carbon content and the total number of features in each sampling site.

Table S7. Provincial environmental and socioeconomic data for preliminary correlation with micropollutant concentrations. (Please see more details in uploaded excel file).

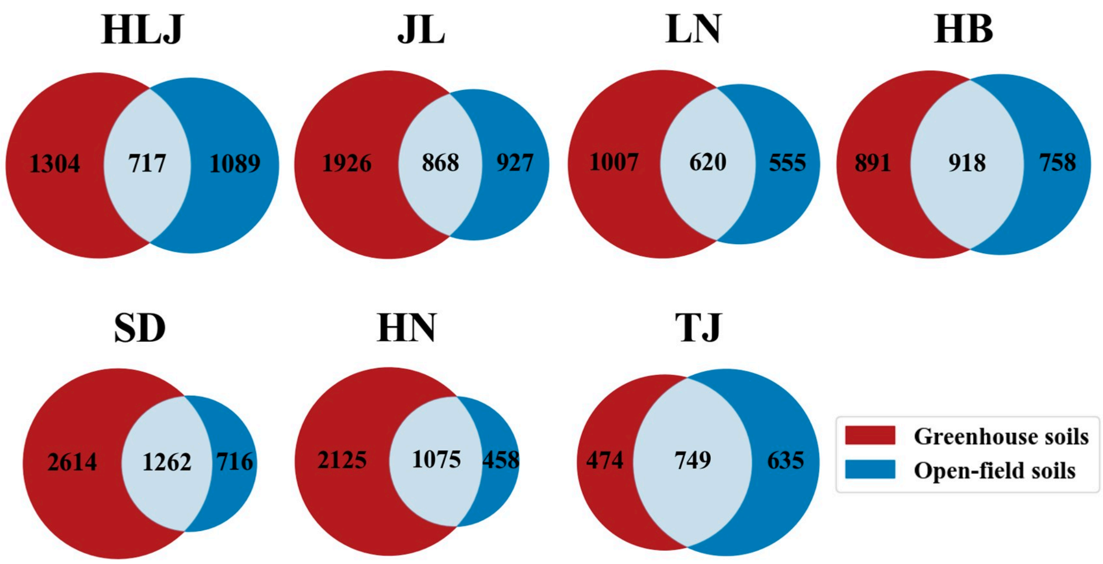

Figure S5. Distribution of regional features in greenhouse and open-field soils.

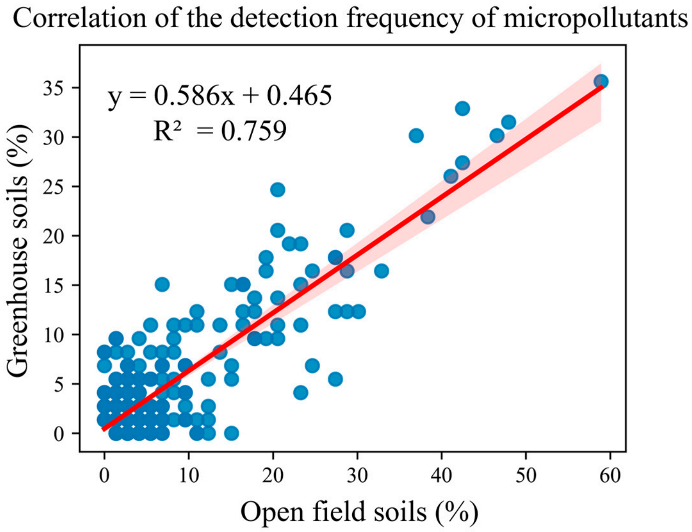

Figure S6. Pearson correlation of detection frequencies (DFs) of micropollutants (n = 498) in greenhouse and open-field soils.

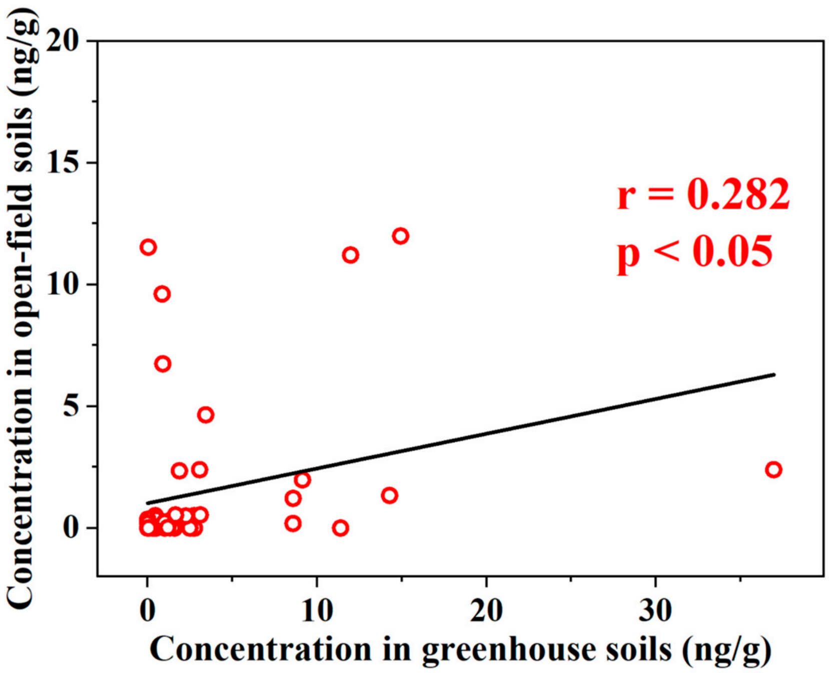

Figure S7. Correlation between micropollutant concentrations between greenhouse soils and open-field soils.

Table S8 Risk quotients (*RQ*s) for micropollutants in 73 soil samples (Please see more details in uploaded excel file).

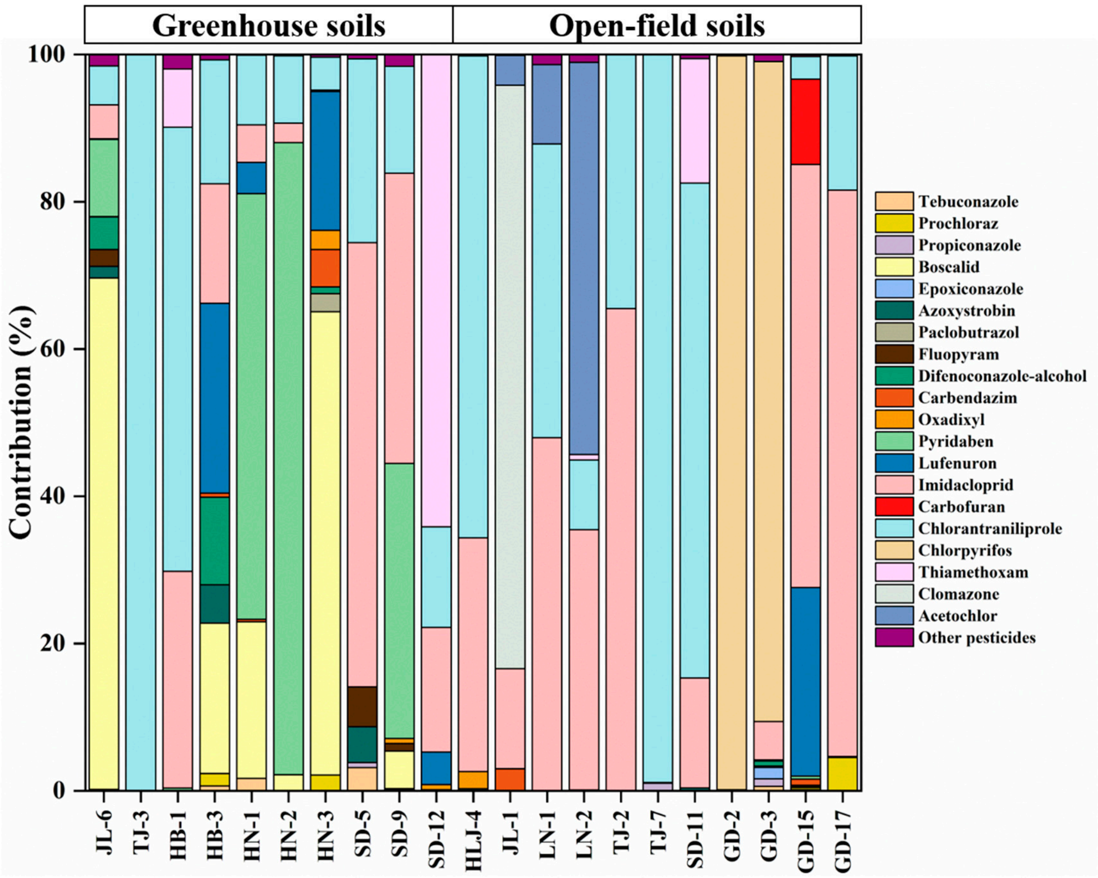

Figure S8 Relative contribution of individual pesticides to the overall risk at 21 high-risk sites ($\Sigma RQ_{\text{site}} \geq 1$).

References

Bhandari, G., Atreya, K., Scheepers, P. T. J., & Geissen, V. (2020). Concentration and distribution of pesticide residues in soil: Non-dietary human health risk assessment. *Chemosphere*, *253*. https://doi.org/10.1016/j.chemosphere.2020.126594

Froger, C., Jolivet, C., Budzinski, H., Pierdet, M., Caria, G., Saby, N. P. A., Arrouays, D., & Bispo, A. (2023). Pesticide Residues in French Soils: Occurrence, Risks, and Persistence. *Environmental Science & Technology*, *57*(20), 7818-7827. https://doi.org/10.1021/acs.est.2c09591

Guo, S. Z., Zhang, S. H., Zhang, J. J., Lei, J., & Chen, H. Z. (2025). Occurrence, residue level, distribution and risk assessment of pesticides in the typical polder areas of Lake Dongting. *Journal of Hazardous Materials*, *496*. https://doi.org/10.1016/j.jhazmat.2025.139530

Hagner, M., Rämö, S., Soinne, H., Nuutinen, V., Muilu-Mäkelä, R., Heikkinen, J., Hyvönen, J., Ohralahti, K., Silva, V., Osman, R., Geissen, V., Ritsema, C. J., & Keskinen, R. (2024). Pesticide residues in boreal arable soils: Countrywide study of occurrence and risks. *Environmental Pollution*, *357*. https://doi.org/10.1016/j.envpol.2024.124430

Hvezdová, M., Kosubová, P., Kosíková, M., Scherr, K. E., Simek, Z., Brodsky, L., Sudoma, M., Skulcová, L., Sánka, M., Svobodová, M., Krkosková, L., Vasícková, J., Neuwirthová, N., Bielská, L., & Hofman, J. (2018). Currently and recently used pesticides in Central European arable soils. *Science of the Total Environment*, *613*, 361-370. https://doi.org/10.1016/j.scitotenv.2017.09.049

Li, H. F., Gong, W. W., Lv, W. X., Wang, Y. R., Dong, W. T., & Lu, A. X. (2023). Target and suspect screening of pesticide residues in soil samples from peach orchards using liquid chromatography quadrupole time-of-flight mass spectrometry. *Ecotoxicology and Environmental Safety*, *253*, 114664. https://doi.org/10.1016/j.ecoenv.2023.114664

Riedo, J., Wettstein, F. E., Rösch, A., Herzog, C., Banerjee, S., Büchi, L., Charles, R., Wächter, D., Martin-Laurent, F., Bucheli, T. D., Walder, F., & van der Heijden, M. G. A. (2021). Widespread Occurrence of Pesticides in Organically Managed Agricultural Soils-the Ghost of a Conventional Agricultural Past? *Environmental Science & Technology*, *55*(5), 2919-2928. https://doi.org/10.1021/acs.est.0c06405

Silva, V., Mol, H. G. J., Zomer, P., Tienstra, M., Ritsema, C. J., & Geissen, V. (2019). Pesticide residues in European agricultural soils - A hidden reality unfolded. *Science of the Total Environment*, *653*, 1532-1545. https://doi.org/10.1016/j.scitotenv.2018.10.441

Wang, W. Q., Wang, D. H., Liu, Q. Z., Lin, L. H., Xie, Y. C., & Du, C. (2024). Distribution Characteristics and Risk Assessment of 57 Pesticides in Farmland Soil and the Surrounding Water. *Toxics*, *12*(1).

https://doi.org/10.3390/toxics12010085
